# Osgood-Schlatter Disease as a Possible Cause of Tibial Tuberosity Avulsion

**DOI:** 10.7759/cureus.13256

**Published:** 2021-02-10

**Authors:** Brandon M Carius, Brit Long

**Affiliations:** 1 Emergency Medicine, Brian D. Allgood Army Community Hospital, Camp Humphreys, KOR; 2 Emergency Medicine, Brooke Army Medical Center, Fort Sam Houston, USA

**Keywords:** knee injuries, knee trauma, pediatric fractures

## Abstract

Osgood-Schlatter disease (OSD) proposes that bony microtrauma of the patellar tendon insertion on the tibial tuberosity may be due to inappropriate stress with adolescent activity, and is a common pathology among pediatric patients. Lack of activity restrictions may further contribute to significant bony damage due to continued quadriceps contraction, which in some cases results in a tibial tuberosity avulsion fracture. Evaluation in the ED should include distal neurovascular status, as compartment syndrome has also been documented. Radiographs are generally definitive for diagnosis; however, bedside ultrasound and CT may help further define injury severity and delineate conservative rather than operative management. We highlight the case of a 13-year-old male with a recently diagnosed history of OSD who presented to the ED for severe knee pain after landing forcefully onto the ipsilateral foot and was found to have a large avulsion fracture of the tibial tuberosity. We also provide a brief review of the literature.

## Introduction

Osgood-Schlatter disease (OSD) describes a bony outgrowth resulting from repetitive microtrauma to the tibial tubercle in the adolescent population [1,2]. While this condition is overwhelmingly managed conservatively with activity restrictions and analgesics, noncompliance and further undue stress may create complications, including tibial tuberosity avulsion fractures. Here, we describe the case of a pediatric patient with recently diagnosed OSD presenting to the ED with workup revealing a large avulsion fracture of the tibial tuberosity.

## Case presentation

**Figure 1:**
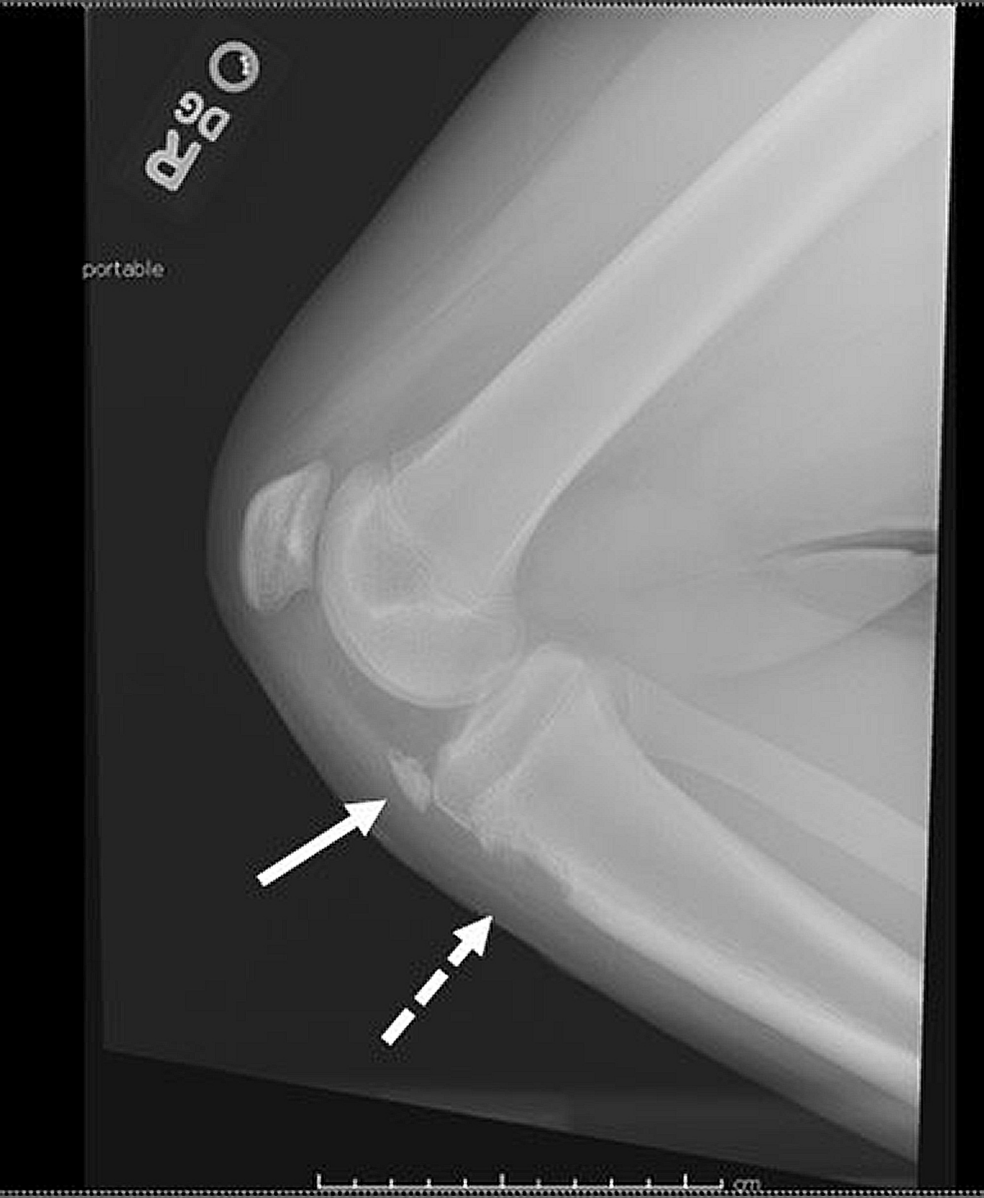
Lateral radiograph demonstrates a 21x9 mm bony fragment (solid arrow) retracted approximately 4.5 cm from the tibial tuberosity (dotted arrow).

**Figure 2:**
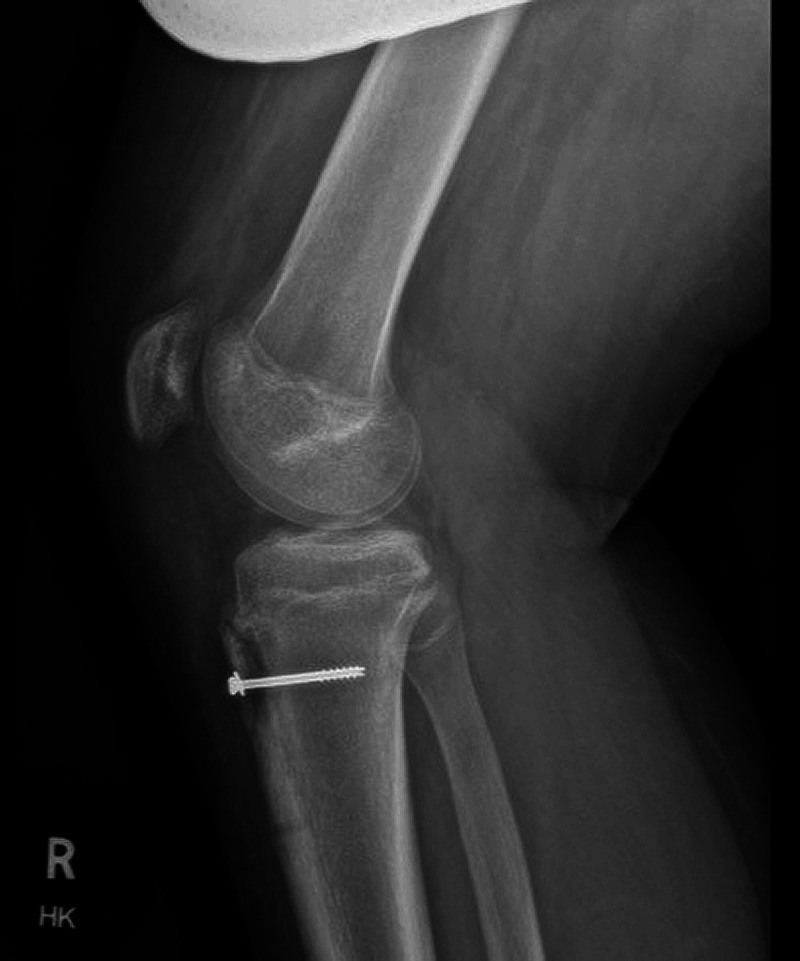
Postoperative lateral radiograph demonstrates an isolated screw securing the bone fragment to the tibia.

A 13-year-old morbidly obese male (BMI 36) with a history of OSD presented to the ED via ambulance for acute onset of severe right knee pain and inability to move the knee from a flexed position. The patient briefly described the mechanism of his injury as jumping off a short wall estimated at a height of 3 feet and landing on his right foot only, after which his knee immediately gave way at the onset of pain. He denied any distal numbness, tingling, or any other injuries or pain. Physical examination was remarkable for a grossly deformed right knee, held in a 90-degree flexed position, with the patella superior and lateral to its normal lie and severe tenderness around the proximal anterior tibia without a palpable patellar tendon. Pulses were palpable and neurological examination was normal distal to the injured knee. The lower leg compartments were soft and nontender. Bedside ultrasound demonstrated an intact patellar tendon without apparent tibial insertion and lack of mobility on attempted active knee extension. Plain radiographs of the right knee revealed a patellar tendon avulsion fracture of the tibial tubercle, with a 21x9 mm fragment retracted approximately 4.5 cm from the tibial tuberosity resulting in patella alta (Figure 1). Orthopedic consultation resulted in admission for operative repair and screw fixation of the tibial fragment (Figure 2).

## Discussion

Robert Bayley Osgood and Carl B. Schlatter independently described chronic microtrauma of the adolescent tibial tubercle in 1903 [1,2]. The proposed mechanism for the development of painful bony nodules at the patellar tendon insertion was a repetitive injury from a forceful contraction of the quadriceps muscle during jumping and sprinting movements [2]. The prevalence of this condition among the adolescent population is estimated to be approximately 9.8%, with a male predominance [3]. Following diagnosis, activity restrictions are recommended to allow for proper healing and avoid possible complications, including tibial tuberosity fracture and compartment syndrome [2-6]. While tuberosity avulsion fractures make up only 3% of all proximal tibial fractures, and generally less than 1% of all physeal injuries, the frequency of this complication of OSD is not well defined in the literature [4-6]. OSD is nevertheless frequently discussed as a predisposing factor for these injuries, especially in adolescents with continued activity against recommended restrictions [2,4,6-10]. Case series demonstrating 20-75% of adolescents with tibial avulsion fractures were previously diagnosed with OSD [7,10].

Like OSD, tibial tuberosity avulsion fractures result from a sudden and violent contraction of quadriceps muscles placing severe strain on the patellar tendon which overcomes physeal strength [2,4,5,6,11]. This mechanism becomes more prevalent in later years of adolescence [8,12]. Structural weakening of the physical cartilage secondary to repetitive stress in OSD, along with fibrocartilaginous maturation of the patellar tendon, may therefore risk avulsion injury [2,4,13]. Literature suggests that the time period between OSD diagnosis and avulsion fracture can range from three weeks or up to one year prior to fracture, but individual cases do not consistently discuss OSD severity or specify the level of activity leading up to injury [2,11,14-16].

Assessment in the ED setting should focus on immediate stabilization and assessment for other injuries and secondary complications to include distal neurovascular examination on the affected limb. A thorough evaluation is paramount since disruption of the anterior tibial recurrent artery may lead to compartment syndrome in rare cases [4,5,11,17,18]. Bedside ultrasound can be employed for tendinous and soft tissue evaluation, as documented cases illustrate simultaneous injuries of the patellar and quadriceps tendons [5,11]. Plain radiographs are generally definitive for diagnosis, and some orthopedic experts advocate for imaging with the knee in 90-degrees flexion and full extension to evaluate the degree of displacement [19]. CT may be required for orthopedic categorization and surgical planning [5,11]. MRI can be considered to evaluate for ligamentous injury, however this is unlikely to not change the immediate management [7,11,14].

Minimal displacement of the avulsed fragment can be considered for limb immobilization, though there is limited supporting literature compared to surgical fixation [5,7,8,11]. Displaced fragments or other complications necessitate orthopedic consultation for open reduction and internal fixation, generally with strong outcomes [5,7,11]. Only one patient is documented to have developed postoperative deep venous thrombosis, but extended limb mobility should be considered as a risk during recovery [7].

## Conclusions

Without adherence to activity limitations, continued tibial tuberosity microtrauma can lead to bony avulsion in pediatric populations. Emergent evaluation focuses on distal neurovascular status, as compartment syndrome can occur. Conservative management with limb immobilization is an option, however surgical fixation demonstrates improved outcomes. This patient's postoperative course was initially complicated by poor bone healing, but improved with physical therapy engagement.
